# The Validity of Claims-Based Algorithms to Identify Serious Hypersensitivity Reactions and Osteonecrosis of the Jaw

**DOI:** 10.1371/journal.pone.0131601

**Published:** 2015-07-10

**Authors:** Nicole C. Wright, Jeffrey R. Curtis, Tarun Arora, Wilson K. Smith, Meredith L. Kilgore, Kenneth G. Saag, Monika M. Safford, Elizabeth S. Delzell

**Affiliations:** 1 Department of Epidemiology, University of Alabama at Birmingham, Birmingham, Alabama, United States of America; 2 Department of Medicine, Division of Clinical Immunology & Rheumatology, University of Alabama at Birmingham, Birmingham, Alabama, United States of America; 3 Department of Health Care Organization & Policy, University of Alabama at Birmingham, Birmingham, Alabama, United States of America; 4 Department of Medicine, Division of Preventive Medicine. University of Alabama at Birmingham, Birmingham, Alabama, United States of America; University of North Carolina at Chapel Hill, UNITED STATES

## Abstract

Validation of claims-based algorithms to identify serious hypersensitivity reactions and osteonecrosis of the jaw has not been performed in large osteoporosis populations. The objective of this project is to estimate the positive predictive value of the claims-based algorithms in older women with osteoporosis enrolled in Medicare. Using the 2006-2008 Medicare 5% sample data, we identified potential hypersensitivity and osteonecrosis of the jaw cases based on ICD-9 diagnosis codes. Potential hypersensitivity cases had a 995.0, 995.2, or 995.3 diagnosis code on emergency department or inpatient claims. Potential osteonecrosis of the jaw cases had ≥1 inpatient or outpatient physician claim with a 522.7, 526.4, 526.5, or 733.45 diagnosis code or ≥2 claims of any type with a 526.9 diagnosis code. All retrieved records were redacted and reviewed by experts to determine case status: confirmed, not confirmed, or insufficient information. We calculated the positive predictive value as the number of confirmed cases divided by the total number of retrieved records with sufficient information. We requested 412 potential hypersensitivity and 304 potential osteonecrosis of the jaw records and received 174 (42%) and 84 (28%) records respectively. Of 84 potential osteonecrosis of the jaw cases, 6 were confirmed, resulting in a positive predictive value (95% CI) of 7.1% (2.7, 14.9). Of 174 retrieved potential hypersensitivity records, 95 were confirmed. After exclusion of 25 records with insufficient information for case determination, the overall positive predictive value (95% CI) for hypersensitivity reactions was 76.0% (67.5, 83.2). In a random sample of Medicare data, a claim-based algorithm to identify serious hypersensitivity reactions performed well. An algorithm for osteonecrosis of the jaw did not, partly due to the inclusion of diagnosis codes that are not specific for osteoporosis of the jaw.

## Introduction

Osteoporosis (OP) is an important public health problem affecting millions of older Americans [1]. Treatments include a variety of agents, while the most prescribed class of drugs is the bisphosphonates. Adverse events listed in product packaging for patients initiating bisphosphonate therapy include esophageal and stomach irritation/pain for the oral agents, and headache, constipation, diarrhea, and muscle/joint pain for intravenous preparations [2]. The safety of long-term use of bisphosphonates has been questioned recently as higher rates of fractures at the subtrochanteric and diaphyseal regions of the femur [3–5], known in the field as atypical femoral fractures, and osteonecrosis of the jaw (ONJ) [6–9] have been found.

In 2004, the United States Surgeon General identified a number of remaining knowledge gaps, one of which is tracking the occurrence of adverse events that might be associated with the various OP medications, particularly in the post-market setting [10]. Given this gap and the increasing recognition of new safety concerns in medications, such as atypical femoral fractures and ONJ in patients using OP medications, regulatory agencies have called for large-scale post-marketing surveillance studies of all newly marketed OP medications.

An example of a recently approved osteoporosis agent with FDA mandatory post-marketing safety evaluation is Prolia (denosumab 60 mg), approved in May of 2010. Unlike bisphosphonates, Prolia targets the receptor activator of nuclear factor kappa-B (RANK) ligand to suppress bone resorption [11], thus making the safety profile potentially different than that of bisphosphonates. In the FDA mandate, the makers of Prolia were charged with evaluating the adverse events associated with bisphosphonate use as well as those found in the phase-III Prolia clinical trials. It is clear that post-marketing safety studies are becoming the way of the future. Analyzing methods to best assess post-marketing safety is essential.

Administrative data from large commercial and non-commercial insurers have been used to conduct postmarking surveillance studies because of the size of their database and comprehensive collection of information on drug exposure and outcomes of interest. However, in order for results to be considered valid, the adverse events of interest need to be ascertained from administrative claims data by algorithms with high positive predictive values (PPVs). The goal of this study was to evaluate the validity of claims-based algorithms’ identification of true cases of two of the adverse events of interest in a nationwide sample of postmenopausal women with OP in the United States Medicare system: 1) ONJ and 2) hypersensitivity reactions leading to hospitalization or hospital emergency department (ED) visit.

## Methods

### Data Source and Population

We conducted a retrospective analysis of data on the national 5% sample of Medicare beneficiaries. Data included demographic information, all medical services claims and Part D prescription events data from 2006–2008. We identified women with postmenopausal osteoporosis (PMO) based on an age criterion consistent with menopausal status (≥ 65 years old) and using an algorithm based upon osteoporosis diagnosis codes, fragility fracture diagnosis and repair codes, and osteoporosis medication codes (data in S1 Text). In order to evaluate incident events, women were eligible to participate in the study if they had 13 consecutive months of traditional fee-for-services Medicare (Parts A, B and not enrolled in a Medicare Advantage Plan) and prescription coverage (Part D). Women with claims for either condition in the 12 months prior to the PMO index date (baseline period) were excluded.

### Selection of Potential Cases

#### ONJ

We identified potential cases of ONJ using inpatient, outpatient, and physician visit claims with the International Classification of Diseases, 9^th^ revision (ICD-9) diagnosis codes 733.45, 522.7, 526.4, 526.5, and 526.9 Table 1. With the exception of 526.9, all of the codes have been used in previous studies evaluating ONJ [6, 8, 9, 12]. We added 526.9 to increase the number of potential cases. Due to the non-specific nature of the 526.9 code, beneficiaries were considered potential cases only if they had ≥2 inpatient, outpatient, or physician visit claims with this code.

**Table 1:** Translation of International Classification of Disease 9^th^ Edition (ICD-9) Codes.

ICD-9 code	Description
**ONJ**	
522.7	Periapical abscess with sinus
526.4	Inflammatory conditions of jaw
526.5	Alveolitis of jaw
526.9	Jaw pain, Not otherwise specified
733.45	Aseptic necrosis of bone, jaw
**Hypersensitivity**	
288.3	Eosinophilia
713.6	Arthropathy associated with hypersensitivity reaction
995.0	Other anaphylactic reaction
995.2	Unspecified adverse effect of unspecified drug, medicinal and biological substance not elsewhere classified
995.3	Allergy, unspecified, not elsewhere classified

#### Hypersensitivity

We identified potential hypersensitivity cases using ICD-9 codes 288.3, 713.6, 995.0, 995.2, and 995.3 Table 1. Women were only considered as potential cases if the claim included a primary hospital discharge diagnosis or the claim had a primary hypersensitivity related diagnosis on an ED claim. Claims were identified as being ED visits if they had Healthcare Common Procedure Coding System (HCPCS) codes or place of service code on the claims that indicated the ED.

Using these algorithms, we selected all potential cases of ONJ (n = 573) and a sample of 568 potential cases of hypersensitivity from a total of 1,033. We chose these sample sizes under the assumption that they would yield 100 participants with potential ONJ and 200 participants with potential hypersensitivity.

### Acquiring Beneficiary Contact Information

We submitted encrypted beneficiary identification numbers for the selected potential ONJ and hypersensitivity cases to CMS’ data center to link the encrypted identifier to the name and address information for each potential case. After receiving the contact information from CMS, the Beneficiary Contact Service (BCS) at University of Minnesota sent an initial notification letter to the beneficiaries about participation in our study. After a three week response period, we received the contact information for the beneficiaries who specified interest in the study or who did not respond to the BCS letter.

### Acquiring Provider Contact Information & Authorization Requirements

We linked provider identifiers in the claims data to the Medicare Place of Service file to identify hospital contact information and to the American Medical Association Provider File and Medicare Physician Identification and Eligibility Registry to identify physician contact information. Information Collection Enterprise, LLC (ICE), the medical records collection agency used for the project, contacted the providers by mail to determine the specific documents needed for authorization of release of medical records.

### Solicitation of Medical Records

Based on our approved protocol from the University of Alabama at Birmingham’s Institutional Review Board (UAB IRB), we mailed a study packet to all potential cases including specific study information and medical records release authorization form (generic or provider specific) to obtain written consent to retrieve medical records. Packets were re-mailed to all beneficiaries two weeks after first mailing. For those agreeing to participate, ICE contacted providers to obtain medical records. All consenting participants received a $25 gift card as compensation for their time in the study.

We modified our medical record solicitation process to seek release of medical records from providers directly, without beneficiaries’ authorization. This changed was approved by the CMS Privacy Board based on a HIPAA waiver from the UAB IRB. We mailed providers a letter regarding the objectives of our study along with instructions for providing the pertinent records. ICE sent a follow-up mailing three weeks after the first and also attempted telephone contact with the various physician offices and medical records departments.

### Adjudication

ICE removed all personal identifying information from the records and digitized the redacted records before sending anonymized records to UAB. ONJ was adjudicated by an expert panel consisting of dentists, an oral/maxillofacial surgeon, and researchers in head, neck, and oral oncology. A confirmed case was one in which there was evidence in the record of 1) oral cavity involvement, 2) that there was an area of exposed alveolar or palatal bone for at least weight weeks, 3) that the patient received appropriate care treatment of the exposed bone, and 4) that the patient did not have a history of radiation therapy to the head, face or mouth. These criteria were based on clinical and diagnostic information from groups including the ASBMR taskforce on ONJ and the American Association of Oral and Maxillofacial Surgeon [7, 13]. The adjudicators recorded case determination of the anonymized records on a standardized case report forms as confirmed or not confirmed.

Physicians at the UAB Department of Dermatology reviewed the hypersensitivity records, evaluating: 1) the presence of hypersensitivity symptoms, 2) symptom onset, and 3) potential triggers of symptoms. Hypersensitivity symptoms of interest included those classified as Type I reactions in the Gell and Coombs Classification of Drug Hypersensitivity reactions, including urticaria, angioedema, bronchospasm, pruritus, vomiting, diarrhea, and anaphylaxis [14]. A confirmed case was one in which there was evidence of acute onset of one of the hypersensitivity symptoms. Adjudicators recorded case status (confirmed, non-confirmed, and indeterminate) of the anonymized records on case report forms.

### Statistical Analysis

We calculated the retrieval rate overall, by ICD-9 code and provider type (hospital vs. physician office for ONJ and inpatient vs. ED for hypersensitivity). We calculated the positive predictive value (PPV) of the claims-based algorithm as the number of definite cases confirmed by medical review divided by the number of potential cases for which records with sufficient information were obtained. We calculated ICD-9 specific PPVs for ONJ, and separately for anaphylaxis (995.0) and other hypersensitivity (288.3, 713.6, 995.2, 995.3) combined. We computed descriptive statistics by confirmation status to evaluate demographic and medical differences between the confirmation groups.

## Results

We identified 202,324 women with PMO (12% of the 2006–2008 5% sample) that had at least 13 months of continuous traditional fee-for-service Medicare coverage without being enrolled in a Medicare Advantage Plan. Our validation study included 1,141 women from the PMO cohort: all potential ONJ cases (n = 573) and the sample of potential hypersensitivity cases (n = 568). The potential ONJ cases included 29 (5%) women identified with diagnosis code 522.7, 335 (59%) with 526.4, 11 (2%) with 526.5, 168 (29%) with 526.9, and 30 (5%) with the specific ONJ code 733.45. Of the selected 568 potential hypersensitivity cases, 38 (6.7%) were identified with a 995.0 diagnosis code, 103 (18.1%) with 995.2, and 427 (75.2%) with diagnosis code of 995.3. None of the selected potential cases had 288.3 or 713.6 diagnosis codes.

**Fig 1:**
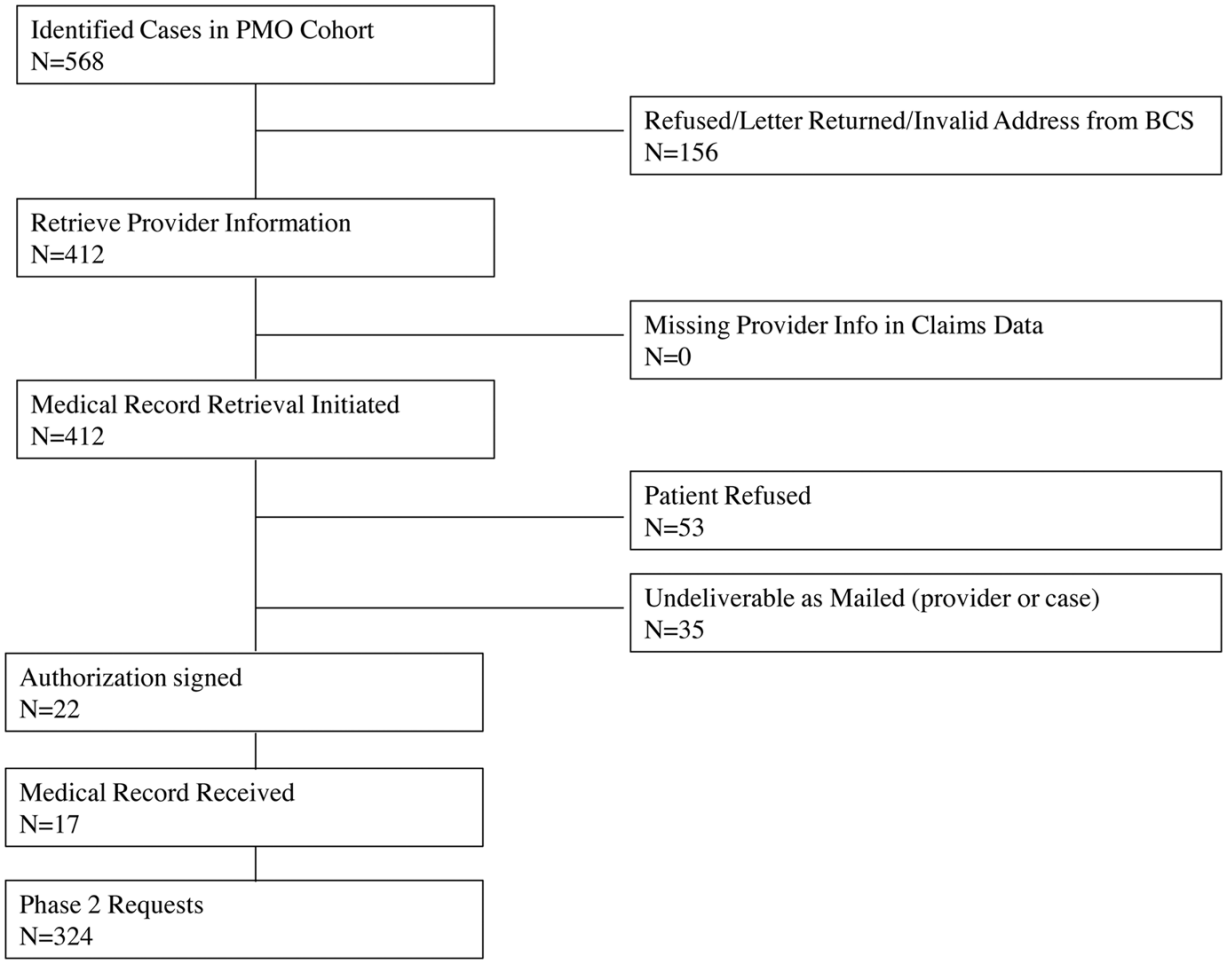
US Medicare Participant Selection Flowchart—ONJ.

**Fig 2:**
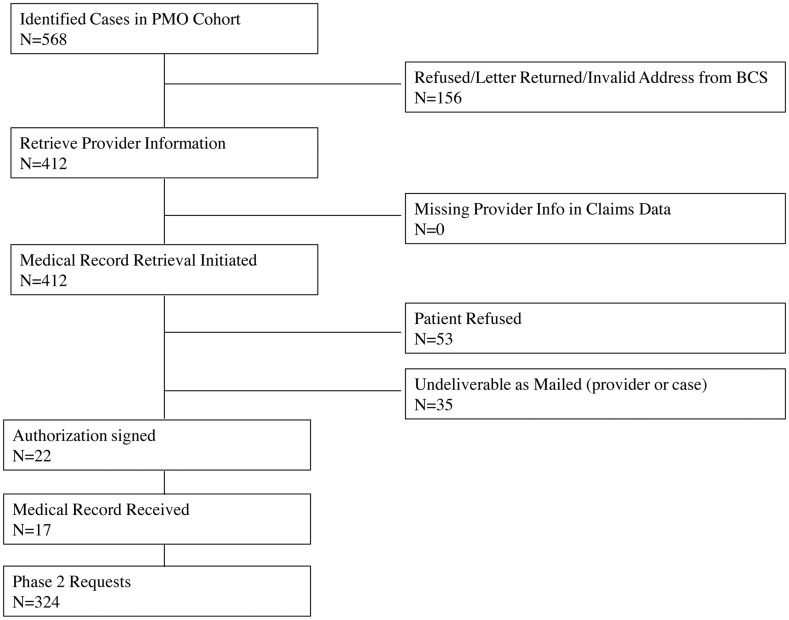
US Medicare Participant Selection—Hypersensitivity.

Figs 1 and 2 describe the medical records solicitation process for both conditions. We excluded 307 potential cases (ONJ: 151 “Fig 1”; hypersensitivity: 156 “Fig 2”) for lack of contact information from BCS. Of those remaining, we were unable to retrieve provider information for another 118 potential cases, all of which were potential ONJ cases (Figs 1 and 2). We initiated medical record retrieval on 304 potential ONJ cases and 412 potential hypersensitivity cases. Only 10 (3%) potential ONJ and 22 (5%) potential hypersensitivity cases signed the authorization form to release their medical records (Figs 1 and 2). Thus, we implemented phase 2.

Phase 2 included a total of 248 requests to providers for records of potential ONJ cases and 324 requests for records of potential hypersensitivity cases. We received records for a total of 84 (27.6% of 304) potential cases with ONJ Table 2. We received 41 (61%) of 67 records requested from hospital providers, whereas we received only 43 (18%) of the 237 records requested from physician offices. Overall, we received 174 (42.2% of 412) records from patients with a potential hypersensitivity reaction Table 2. The vast majority of hypersensitivity cases were identified from ED claims (n = 398), of which we received 164 (41%) of those requested. We requested 14 potential hypersensitivity records from inpatient settings, of which we received 10 (71%).

**Table 2:** Proportion of Successfully Retrieved Medical Records by Facility Type.

	Overall		Hospital		Physician Office^a^/ER^b^	
	Requested (n)	Obtained, n (% Retrieved)	Requested (n)	Obtained, n (% Retrieved)	Requested (n)	Obtained, n (% Retrieved)
**ONJ**	**304**	**84 (27.6)**	**67**	**41 (61.2)**	**237**	**43 (18.1)**
522.7	13	2 (15.4)	0	0 (0.0)	13	2 (15.4)
526.4	166	38 (22.9)	12	7 (58.3)	154	31 (20.1)
526.5	4	2 (50.0)	1	1 (100.0)	3	1 (33.3)
526.9	112	38 (33.9)	52	30 (59.6)	60	7 (11.7)
733.45	9	4 (44.4)	2	2 (100.0)	7	2 (28.6)
**Hypersensitivity**	**412**	**174 (42.2)**	**14**	**10 (71.4)**	**398**	**164 (41.2)**
995.0	28	16 (57.1)	10	6 (60.0)	18	10 (55.6)
995.2 & 995.3	384	158 (41.1)	4	4 (100.0)	380	154 (40.5)

^a^ONJ

^b^Hypersensitivity

We observed significant differences in the mean age of the selected, contacted, and record received potential ONJ cases, with older ages among the women we selected and received records than those we contacted for retrieval Table 3. We also received a significantly larger proportion (16.1%) of potential hypersensitivity records from African Americans than we initially selected (3.2%) or contacted (6.7%) for retrieval Table 3. We heard from 101 providers (33.2%) of potential ONJ cases and 112 providers of (27.2%) hypersensitivity cases indicating reasons why records could not be released, which were primarily related to the need for patient consent or the inability to locate requested records.

**Table 3:** Characteristics of Potential Cases by Contact Status.

	ONJ				Hypersensitivity			
	Selected	Contacted^a^	Received	p-value	Selected	Contacted^a^	Received	p-value
**Age (mean, SD)**	77.8 (7.8)	76.9 (8.4)	77.0 (7.4)	0.018	78.0 (7.9)	75.0 (6.9)	76.7 (7.8)	<0.001
**Race/Ethnicity (%)**				0.383				0.003
White	91.8	86.4	92.9		89.1	86.6	77.6	
Black	2.2	5.5	3.6		3.2	6.7	16.1	
Hispanic	2.2	3.6	1.2		4.5	3.4	4.0	
Other	3.7	4.6	2.4		3.2	3.4	2.3	
**Case Year (%)**				0.348				0.867
2006	32.0	37.3	33.3		49.4	51.3	50.6	
2007	33.7	33.2	40.5		28.9	25.2	24.1	
2008	35.3	29.6	26.2		21.8	23.5	25.3	
**Region (%)**				0.012				0.762
Northeast	17.8	30.0	14.3		17.3	23.1	18.4	
Midwest	31.6	22.7	32.1		23.1	18.5	20.1	
South	35.7	30.9	39.3		48.1	46.2	47.7	
West	14.9	16.4	14.3		11.5	12.2	13.8	

^a^ Contacted during Phase1 or Phase 2

Table 2 provides the distribution of requested and retrieved records by ICD-9 diagnosis code. The largest proportion of retrieved ONJ records came from claims with a 526.5 diagnosis code (2 of 4; 50.0%) and the specific ONJ diagnosis code of 733.45 (4 of 9; 44.4%), followed by non-specific jaw pain 526.9 (38 of 112; 33.9%), 526.4 (38 of 166; 22.9%), and finally 522.7 (2 of 13; 15.4%). For potential hypersensitivity cases, we received a larger proportion of records associated with claims having anaphylaxis as the primary diagnosis code (57.1%) compared to the remaining hypersensitivity codes (41.1%).

Six ONJ cases were confirmed and 78 cases were adjudicated as a non-case, resulting in an overall PPV (95% CI) of 7.1% (2.7, 14.9). The majority (n = 5) of the confirmed cases had the 526.4 code, which had a PPV (95% CI) of 12.5% (4.2, 26.8). The specific ONJ code (733.45) had one confirmed case out of four, which resulted in the highest ONJ PPV (95% CI) of 25.0% (0.6, 80.6). We found an overall higher PPV (95% CI) for physician office records [11.6% (3.9, 25.1)] compared to the PPV of hospital records [2.4% (0.1, 12.9)]; however all of the confirmed 733.45 cases came from hospitals.

Characteristics of confirmed ONJ (n = 6) and confirmed non-cases (n = 78) are described in Table 4. A larger proportion of confirmed cases were from 2007 and a smaller proportion of confirmed cases were from 2008 than those adjudicated as a non-case. The ONJ diagnosis code was in the primary position for 100% confirmed cases, whereas only 28% of confirmed non-cases had the ONJ diagnosis code in the primary position. The top specialty among the confirmed cases was maxillofacial and oral surgery.

**Table 4:** Comparison of Characteristics by ONJ Case Determination Status.

	Confirmed	Not Confirmed	p-value
**Age (Mean, SD)**	85.3 (4.7)	76.3 (7.4)	0.004
**White (%)**	100.0	92.3	0.481
**Case Year (%)**			0.689
2006	33.3	34.6	
2007	50.0	34.6	
2008	16.7	30.8	
**ICD-9 Dx Code (%)**			0.151
522.7	0.0	2.6	
526.4	83.3	44.9	
526.5	0.0	1.3	
526.9	0.0	47.4	
733.45	16.7	3.9	
**Diagnosis Location (%)**			0.130
Primary	100.0	28.2	
Secondary	0.0	71.8	
**Provider Type (%)**			0.102
Hospital	16.7	51.3	
Physician Office	83.3	48.7	
**Physician Specialty (%)**			<0.001
Maxillofacial Surgery	50.0	7.7	
Oral Surgery	16.7	14.1	

Of the 174 potential hypersensitivity cases with medical records, 95 (54.6%) were confirmed, 30 were classified as non-cases (12.7%), and 49 (28.2%) did not have sufficient information to determine case status. For those confirmed as a non-hypersensitivity case, the adjudicators judged that hypersensitivity was not the primary cause of the symptoms. Poor description of symptoms constituted the primary reason for inability to determine cases status.

The overall PPV (95% CI) for the hypersensitivity algorithm after excluding the potential cases with insufficient information was 76.0% (67.5, 83.2). All anaphylaxis records with sufficient information were confirmed (n = 12) resulting in a PPV of 100%. The overall PPV for the remaining hypersensitivity codes combined was 73.5% (64.3, 81.3). Overall, we found a higher PPV (95% CI) for inpatient records [87.5% (47.3, 99.7)] than ER records [75.2% (66.4, 82.7)]. The proportion of hypersensitivity cases with insufficient information was 28.2% (2.6%, 35.5%) overall and did not differ by ICD-9 code.

Characteristics of the hypersensitivity cases by confirmation status are described in Table 5. Race/ethnicity, case year, and provider type were not statistically significantly different among the three case confirmation groups. A larger proportion of those classified as non-hypersensitivity cases had a 995.2 diagnosis code (50% vs. 2.1% in confirmed and 10.2% in unable to determine). Larger proportions of non-hypersensitivity cases (46.4%) and indeterminate cases (55.3%) did not have symptoms that were acute in nature, whereas 56.6% of confirmed cases had a sudden onset of symptoms. The primary symptoms of confirmed cases (84.2%) were related to the skin, including hives and rash compared to only 26.7% in cases that were classified as not hypersensitivity cases.

**Table 5:** Characteristics by Hypersensitivity Case Determination Status.

	Confirmed	Not Confirmed	Indeterminate	p-value
**Age (Mean, SD)**	76.5 (7.9)	79.9 (74.4)	75.3 (7.3)	0.036
**Race/Ethnicity (%)**				0.246
White	73.7	83.3	81.6	
Black	14.7	16.7	16.3	
Hispanic	7.4	0.0	0.0	
Other	4.2	0.0	2.0	
**Case Year (%)**				0.045
2006	43.2	63.3	42.9	
2007	21.1	30.0	26.5	
2008	35.8	6.7	30.6	
**ICD-9 Dx Code (%)**				<0.001
995.0	12.6	0.0	8.2	
995.2	2.1	50.0	10.2	
995.3	85.3	50.0	81.6	
**Record Type (%)**				0.596
Inpatient	7.4	3.3	4.1	
ER	92.6	96.7	95.2	
**Sudden Onset of Symptoms** ^a^ **(%)**				0.001
No	32.5	46.4	55.3	
Yes	56.6	28.6	21.3	
Unknown	10.8	25.0	23.4	
**Symptoms (%)**				
Neurologic	3.2	36.7	8.2	<0.001
Ocular	6.3	0.0	0.0	0.075
Upper Airway	3.2	3.3	2.0	0.918
Lower Airway	10.5	3.3	14.3	0.299
Cardiovascular	8.4	3.3	4.1	0.455
Skin	84.2	26.7	77.6	<0.001
GI	1.1	6.7	6.1	0.163
**Treated by Steroids (%)**	86.3	37.9	71.4	<0.001
Oral	31.7	63.6	62.9	
IV	34.2	18.2	22.9	
Oral + IV	34.2	18.2	14.3	

^a^16 cases were adjudicated on version1 case report form that did not include this question

## Discussion

We conducted a validation study of claims-based algorithms to identify ONJ and hypersensitivity reactions leading to hospitalization. Our retrieval rate for ONJ records was 27.7% and 42.2% for hypersensitivity records. Our findings show that overall the PPV of the algorithm currently used to identify ONJ is low. The diagnosis codes 522.7, 526.5, 526.9 appear to be too non-specific to identify ONJ. The confirmed cases of our study had diagnosis codes of 733.45 and 526.4, with PPVs of 25% and 12.5%, respectively.

Administrative data have been used previously to examine the association between bisphosphonates and ONJ. In 2008, Cartsos et al. evaluated the mode of bisphosphonate administration (oral vs. intravenous) in a large national insurance plan [6]. At the time of their study, the specific ONJ code (733.45) was not available, so they relied on the 526.4 code for ONJ determination. In our study 83.3% of the confirmed cases of ONJ came from the 526.4 diagnosis code; however, the PPV for this code was only 12.5%.

More recently, an evaluation of the validity of administrative codes to identify ONJ was performed in Denmark and Sweden [15, 16]. In both studies, ICD10 codes were used to identify potential cases from oral and maxillofacial surgery inpatient claims. In Denmark, medical records were obtained for all 60 of the potential cases. After chart review, 19 of the cases were confirmed resulting in a PPV for 32% [16]. In Sweden, medical records were retrieved for 83 of the 87 potential cases. Confirmation status was positive for only 15 of the potential cases, which resulted in an overall PPV of 18% [15]. Our PPV was lower than those found in Denmark and Sweden, most probably due to the use of ICD10 codes, which are more specific than ICD9, and the solicitation of claims from oral and maxillofacial surgery, which is one of the top specialties that provide care to ONJ cases.

Our study was limited by a variety of factors regarding ONJ algorithm validation. First, using the available databases, we were unable to determine the provider contact information for a large number of potential ONJ cases. Secondly, we were only able to retrieve 28% of the requested medical records even after we modified our protocol to go directly to providers. Third, we were unable to include an insufficient data category in the final confirmation of ONJ. It is possible that a proportion of the records received did not really have enough information to make a determination, and would have been removed in the estimation of the positive predicted value. Fourth, the adjudicators considered beneficiaries who were exposed to head or neck radiation as a non-case, however, the overall goal of this project was to evaluate the algorithm and not the particular exposure leading to ONJ. If we were to include those cases with head and neck radiation that had all of the other case criteria, although still low, we would have observed a higher overall PPV. Finally, our study was conducted in postmenopausal women with OP, thus our findings may not be generalizable to validation studies conducted in other populations (e.g. cancer patients).

The algorithm used in our study to identify hypersensitivity reactions leading to hospitalization performed moderately well. Hypersensitivity is not a typical reaction of oral bisphosphonates, but has become a concern in newer parenteral OP agents. For example, hypersensitivity has been included as an adverse event in the Prolia package insert [17]. The overall PPV was 76%, and the PPV for claims with the anaphylaxis diagnosis code was 100%.

A recent review of administrative algorithms to identify hypersensitivity reactions and anaphylaxis was performed by Schneider et al. [18, 19]. The studies that included the codes used for our algorithm had PPVs ranging from 1.3%-83.3% [20–23]. Studies that reported results separately for anaphylaxis found PPVs ranging from 52%-57% [20, 21, 24]. A study by Nordstrom et al. also used the three codes of interest in our study and found a combined PPV of 63.6% [22]. The PPVs found in our study were higher than those reported in the review articles, specifically for anaphylaxis. Ongoing work is refining algorithms to potentially improve the PPVs using alternative algorithms.

Again, our results are limited based on the retrieval rate of potential hypersensitivity records. The case report form created for the confirmation of hypersensitivity was based on the clinical acumen of our colleagues in the Department of Dermatology. Our forms may have included different criteria than previous studies, which may explain the differences in PPV values in our study. Lastly, about 25% of the hypersensitivity medical records obtained had insufficient information for case determination. Depending on true case status, the performance of the algorithm could possibly increase or decrease. Similar to ONJ, our findings may not be generalizable to validation studies conducted in other populations.

A major strength of our study is the use of nationwide claims data in a large population of women with postmenopausal osteoporosis of which who had a relatively high exposure to medications that have been associated with the conditions of interest. Utilizing current provider contact databases, conducting more intensive follow-up with the various medical records departments and combining direct-to-provider requests with attempts to obtain patients’ consent to sign records release forms may improve the success rate.

Based on our experience, we recommend that future validation studies of claims-based definitions of ONJ should focus on the specific ONJ diagnosis codes (733.45) as well as the 526.4 code, and that these codes are the primary diagnosis codes on relevant claims. We also recommend that the record retrieval request includes a longer time period (e.g. +/- 90 days) so that adjudicators are able to fully evaluate duration and treatment of symptoms. We also recommend exploring sampling strategies, such as sampling potential cases that have associated factors (i.e. claim from an oral surgeon for ONJ or treatment with steroids for hypersensitivity to) to further improve both algorithms. Analytic methods such as quasi-high dimensional propensity scores can be used to determine these other important associated variables. Currently, we are currently conducting a second validation study, which adds several of these additional elements, to determine its effects on the validity of the algorithms and improve upon them.

Overall, we found moderate to high validity of the claims-based hypersensitivity case identification algorithms and low validity of current claims-based algorithms for ONJ in a sample of older women in Medicare with postmenopausal osteoporosis.

## Supporting Information

S1 Text Algorithm used to identify beneficiaries with postmenopausal osteoporosis. (DOCX)
