# Supplementary material for: The Validity of Claims-Based Algorithms to Identify Serious Hypersensitivity Reactions and Osteonecrosis of the Jaw
Source: PLoS One. 2015 Jul 10;10(7):e0131601. doi: 10.1371/journal.pone.0131601 (PMC4498926; doi:10.1371/journal.pone.0131601)
Supplement: S1 Text — (DOCX) [file pone.0131601.s001.docx]

**Supporting Information**

**Algorithm used to identify beneficiaries with postmenopausal osteoporosis**

- An inpatient primary or secondary diagnosis of osteoporosis
- An inpatient primary or secondary diagnosis of a fracture strongly associated with osteoporosis, including a closed fracture of the hip, spine, pelvis, humerus, femur, or radius/ulna.
- An outpatient (physician office or outpatient clinic) file claim for 1 of the same 6 types of fracture, accompanied by a surgical repair code for the specific type of fracture
- An outpatient (physician office or outpatient clinic) claim for a closed spine fracture with a physician evaluation and management (E/M) HCPCS in the same claim
- An outpatient diagnosis of osteoporosis associated with physician evaluation and management (E/M)
- A claim for an oral osteoporosis medication identified by the National Drug Classification (NDC) brand name, generic name, or therapeutic class, including but not necessarily limited to risedronate (Actonel^®^), branded alendronate (Fosamax^®^), ibandronate [Boniva^®^/Bonviva^®^] oral, generic bisphosphonates including alendronate, neridodronate, and olpadronate, selective estrogen receptor modulators [raloxifene (Evista^®^)], calcitonin, parathyroid hormone analogues [teriparatide (Forteo^®^/Forsteo^®^) and PTH 1-84 (Preotact^®^)], ipriflavone and strontium ranelate
- A claim for an intravenously administered bisphosphonate, including intravenous ibandronate [Boniva^®^/Bonviva^®^] IV, zoledronic acid (Reclast^®^/Aclasta^®^), or pamidronate [Aredia^®^]
